# Maternal PPI therapy during lactation: pantoprazole levels in human milk and possible neonatal implications

**DOI:** 10.1007/s00431-026-07046-7

**Published:** 2026-05-21

**Authors:** Serhat Bor, Pelin Ergun, Selma Sahin, Sezgi Kipcak, Akile Tuncal, Ercument Karasulu

**Affiliations:** 1 Ege Reflux Study Group, Division of Gastroenterology, Ege University School of Medicine, Izmir, Turkey; 2 Department of Otolaryngology and Communication Sciences, Medical College of Wisconsin, Milwaukee, USA; 3 Ege University, ARGEFAR, Izmir, Turkey; 4 Department of Medical Biology, Ege University School of Medicine, Izmir, Turkey; 5 Department of Medical Biochemistry, Cyprus Health and Social Sciences, University School of Medicine, Morphou, Republic of Cyprus

**Keywords:** Pantoprazole, Human breast milk, Breast-feeding, Proton pump inhibitors, Lactation

## Abstract

Proton pump inhibitors (PPIs) are widely used for acid-related disorders, but limited data exist regarding their excretion into human breast milk. This study aimed to quantify pantoprazole levels in plasma and breast milk of lactating women and to assess potential infant exposure. Sixteen mothers who had discontinued breastfeeding and were prescribed 40 mg pantoprazole once daily participated. Blood and breast milk samples were collected on days 1 and 7 at 0, 1.5, 3, 4.5, and 6 h post-dose. Pantoprazole was quantified using a validated high-performance liquid chromatography (HPLC) method with omeprazole as the internal standard. Samples were extracted via liquid–liquid extraction, and the method was validated over a linear range of 0.03–1 µg/mL (LOQ 0.03 µg/mL). Pantoprazole was detected in 46% (69/150) of plasma samples and 17.4% (26/149) of breast milk samples. Plasma levels peaked at ~ 4.5 h, whereas breast milk concentrations peaked at 3–4.5 h on day 1 and at 3 h on day 7. The low detectability and consistently lower breast milk concentrations suggest limited transfer into breast milk. Pantoprazole levels in breast milk were consistently lower than plasma and infrequently detectable.

*Conclusion*: This study, representing the largest series to date examining pantoprazole excretion during lactation, suggests that infant exposure via breast milk is low. These findings suggest that breastfeeding may be compatible with pantoprazole therapy, although caution is warranted. The data provide supportive evidence for clinical decision-making.

**Table:** 

**What is Known:** • *Proton pump inhibitors (PPIs) are widely prescribed during lactation, but robust safety and pharmacokinetic data for pantoprazole transfer into breast milk remain limited.* • *Current breastfeeding recommendations for PPIs are based largely on expert opinion and isolated case reports with limited milk sampling.*
**What is New:** • *Peak concentrations occurred 3–4.5 hours post-dose, and milk levels were consistently much lower than plasma levels.* • *Pantoprazole was detected in few milk samples and at concentrations markedly lower than plasma levels, indicating negligible infant exposure and supporting breastfeeding compatibility during therapy.*

**Graphical Abstract:**

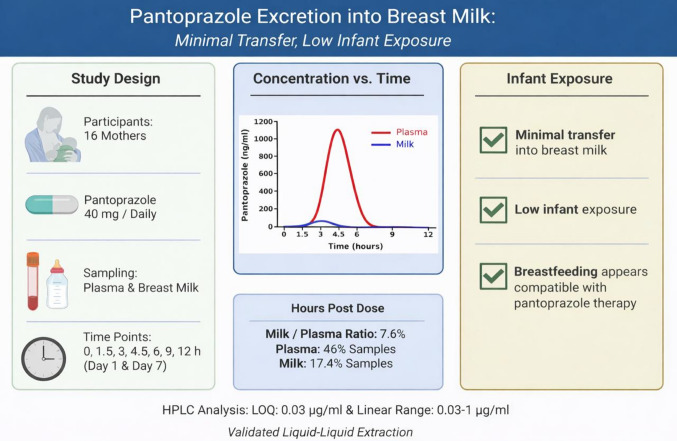

**Supplementary Information:**

The online version contains supplementary material available at 10.1007/s00431-026-07046-7.

## Introduction

Proton pump inhibitors (PPIs) are a cornerstone in the management of acid-related disorders such as gastroesophageal reflux disease (GERD), peptic ulcer disease, and Zollinger–Ellison syndrome [1]. They exert their therapeutic effect by irreversibly inhibiting the hydrogen–potassium adenosine triphosphatase enzyme system in gastric parietal cells, thereby suppressing gastric acid secretion [2]. Pantoprazole, a member of the PPI class, is widely prescribed due to its efficacy, favorable safety profile, and availability in both oral and intravenous formulations [3].

Globally, PPIs are prescribed extensively [4, 5]. A nationwide observational study in France reported 16 million PPI prescriptions in 2015 [6]. In the United States, omeprazole and pantoprazole ranked 10th and 13th, respectively, among the top 200 most commonly prescribed medications in 2023 [7]. In Turkey, annual PPI prescriptions exceed 31 million, representing a substantial healthcare expenditure, and personal PPI use is also high (60.8%), often for inappropriate indications or durations [8]. Despite this widespread use, concerns remain regarding the safety of pantoprazole during breastfeeding. The transfer of medications into breast milk and subsequent exposure to infants is a critical consideration for lactating mothers. While the benefits of breastfeeding are well established, potential risks associated with maternal medication use must be carefully evaluated. Potential concerns include infant exposure through breast milk, possible systemic effects in the infant, and the limited availability of pharmacokinetic and safety data in lactating populations [9].

In terms of pharmacokinetics, pantoprazole undergoes hepatic metabolism primarily via the cytochrome P450 system, especially CYP2C19 and CYP3A4 enzymes [10]. It is eliminated mainly through renal excretion, with a half-life of approximately 1 h in healthy individuals. Importantly, once-daily administration does not result in drug accumulation [11]. The extent of drug excretion into breast milk depends on several factors, including physicochemical properties, protein binding, molecular weight, lipid solubility, and maternal metabolism and excretion rates [9].

Studies directly examining pantoprazole excretion into human breast milk are limited. A case report demonstrated minimal excretion, with an estimated relative infant dose of 7.3 μg following maternal ingestion of 40 mg, suggesting no significant impact on breast milk production or the need for cessation of breastfeeding during chronic use [12].

According to a 2024 ‘Global Breastfeeding Scorecard’ — based on national survey data between 2017–2023 — about 72% of women worldwide continue breastfeeding for at least one year, and nearly half of children are still breastfed at two years, emphasizing that lactation often spans an extended period [13]. Given the extended duration of breastfeeding and the high prevalence of gastroesophageal reflux, investigating PPI transfer into human milk is essential for clinical guidance.

When prescribing pantoprazole or any medication to breastfeeding women, healthcare providers must carefully balance maternal therapeutic benefits against potential risks to the infant. The paucity of data on pantoprazole levels in human breast milk poses challenges for clinical decision-making [14]. This study was therefore designed to address this knowledge gap by systematically investigating pantoprazole concentrations in human breast milk and plasma following oral administration in breastfeeding women. In addition, the study aimed to estimate infant exposure and provide data to better inform clinical practice guidelines for lactating mothers.

## Material and methods

### Human milk sample collection

The study was conducted between 2015 and 2023. Ethical approval was obtained from the Ege University Clinical Research Ethics Committee, Izmir, Türkiye (Approval No: 13—9/27). The study was conducted in accordance with the principles of the Declaration of Helsinki, and all participants provided written informed consent. Participants meeting the criteria shown in Table 1 were included. All mothers had already been prescribed 40 mg pantoprazole once a day by the Ege University, Division of Gastroenterology, Reflux Outpatient. A total of 18 mothers were selected and written informed consent was obtained. The breast milk samples were collected from mothers with full-term babies in the lactation stage, ranging from 14 to 28 months postpartum, who had decided to end lactation. Although participants had decided to discontinue direct breastfeeding, they were still in the lactation phase at the time of enrollment. Lactation was maintained through regular milk expression (manual expression or breast pumping) prior to and during the study period to allow serial sampling. Expressed milk was not used for infant feeding and was discarded.

**Table 1:** The inclusion and exclusion criteria of the subjects

The inclusion criteria	The exclusion criteria
• > 18 years old • Decided to end lactation • Prescribed 40 mg pantoprazole once a day • Being able to spend 12 h in the gastroenterology clinic at day 1 and day 7	• All cancers except nonmelanoma skin cancers, severe or uncontrolled systemic diseases such as chronic obstructive pulmonary disease, coronary artery disease, chronic renal and hepatic disorders, diabetes mellitus • Nonsteroidal anti-inflammatory drug or acetylsalicylic acid usage within last week • Unsigned informed consent form

**Fig. 1:**
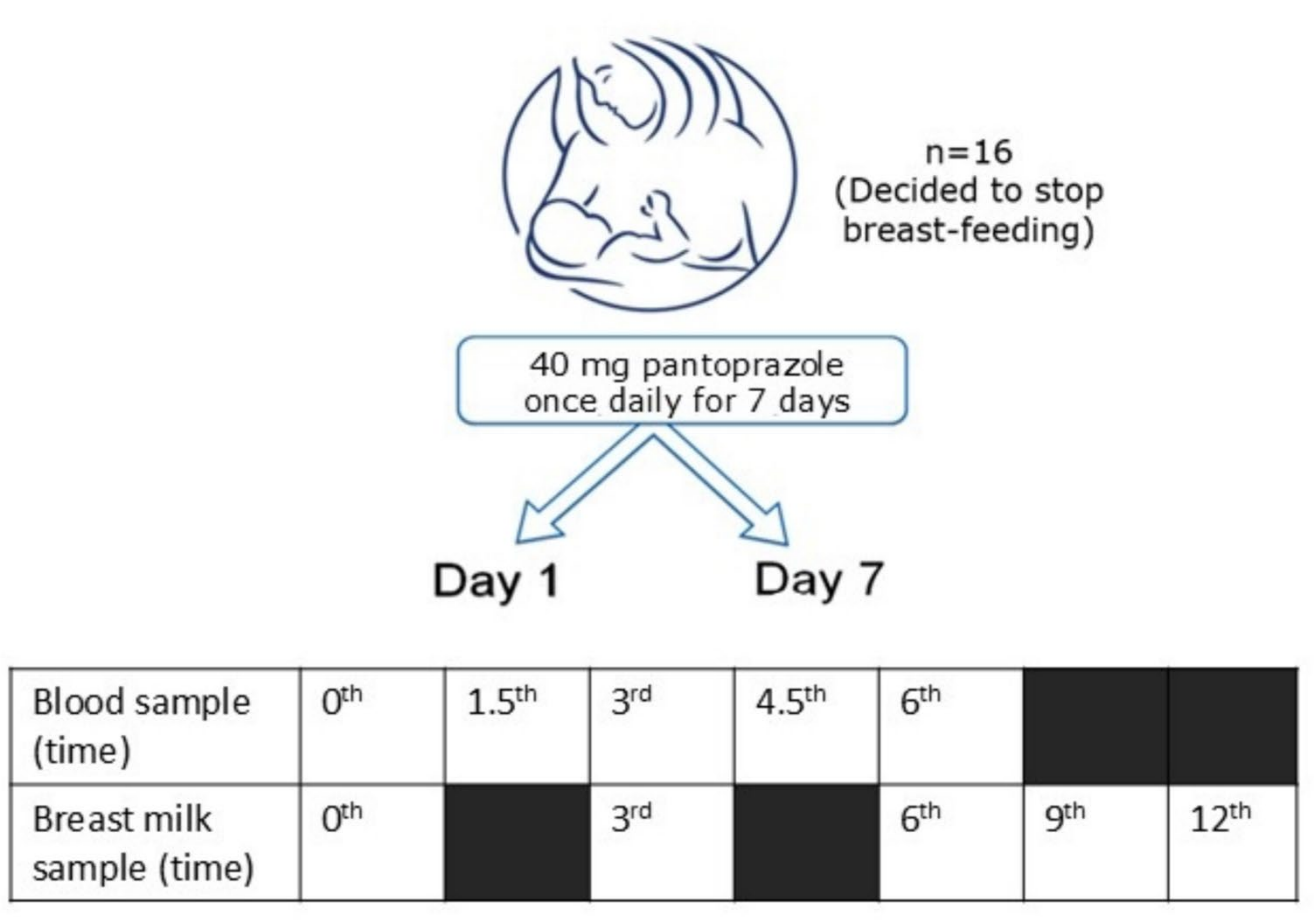
Study Schema. Blood samples were collected on study days 1 and 7 at 0, 1.5, 3, 4.5, and 6 h. The 0-h time point was defined as immediately before the daily pantoprazole dose, following an overnight fast of at least 8 h. Pantoprazole was administered on an empty stomach, and a standardized breakfast was consumed 30 min after PPI intake. Human milk samples were gently expressed from the breast at 0, 3, 6, 9, and 12 h

Blood samples were collected on day 1 and day 7 at the following time points: 0, 1.5, 3, 4.5, and 6 h (Fig. 1). The 0-time point is considered to be just before taking her daily pantoprazole dose on an empty stomach after at least eight hours fasting. The mothers ate their standardized breakfast half an hour after the PPI intake. The selected volunteers carefully cleansed their areola and nipples several times before taking samples and then rinsed relevant parts with sterile water. Human milk was gently expressed from the breast at time points 0, 3, 6, 9, and 12 h post-dose, and the initial drops were discarded. Therefore, the collected samples do not strictly represent foremilk. Approximately 2—3 mL of milk was collected into a 5-mL sterile tube and labeled accordingly. Blood samples, 2 mL in volume, were collected in sterile heparin tubes and labeled. The collected samples were promptly stored at −80 °C until analysis. All samples were collected under controlled clinical conditions.

Pantoprazole has a short elimination half-life (approximately 1 h) and does not accumulate with once-daily dosing [11]; therefore, steady-state conditions are expected to be achieved rapidly, and day 7 sampling was considered representative of steady-state exposure.

The measurements were conducted in 2015, 2017 [15], and 2023 in three sets, using the same device, the same technician, and the same methods, ensuring methodological consistency across all analytical periods. To minimize potential analytical drift, method performance, including calibration, precision, and recovery, was verified during each analytical phase. All samples were stored at − 80 °C until analysis under consistent conditions to preserve sample stability.

### High performance liquid chromatography (HPLC) Analyses

A selective and rapid high-performance liquid chromatography (HPLC) method was developed and validated for quantification of pantoprazole in human plasma and breast milk samples using omeprazole as internal standard. Pantoprazole was extracted from the matrix using a liquid–liquid extraction process. The chromatographic separation was performed on Zorbax Eclipse XDB-C (5 µm, 4.6 × 150 mm) column with a mobile phase consisting of 10 mM potassium dihydrogen phosphate buffer (pH 6) (mobile phase-A) and acetonitrile (mobile phase-B) at a flow rate of 1 mL/min.

The method was validated over a linear concentration range of 0.03 −1 µg/mL and the limit of quantification (LOQ) was 0.03 µg/mL, consistent with previously reported analytical methods [16, 17]. The intra-day and inter-day precision of breast milk samples were expressed as relative standard deviations of 0.563%—5.04% and 3.829%—7.866%, respectively, and those of plasma samples were 0.563%—5.04% and 3.829%—7.866%, respectively. In addition, the recovery of pantoprazole in human plasma and breast milk was found to be between 94—108%.

### Estimation of infant exposure

Estimated infant dose via milk (mg/kg/day) was calculated using a standard milk intake assumption of 150 mL/kg/day [18]. Accordingly, infant exposure was estimated by multiplying the observed milk concentration (mg/L) by 0.15 L/kg/day. Maternal body weight was not prospectively recorded; therefore, maternal dose could not be normalized to mg/kg/day, and formal relative infant dose (RID) calculations were not feasible.

### Statistical analysis

Descriptive statistics were used to summarize pantoprazole concentrations in plasma and breast milk. Concentrations below the lower LOQ were considered non-detectable and were assigned a value of zero for analysis. Estimated infant dose via milk was calculated based on observed milk concentrations using a standard milk intake assumption of 150 mL/kg/day.

## Results

Two of the 18 mothers were excluded from the study due to insufficient breast-milk supply at day 1. The mothers who continued in the study were between 28 and 43 years of age (n = 16) and had discontinued direct breastfeeding but remained in the lactation phase through ongoing milk expression. Table 2 summarizes the demographic characteristics including age, breastfeeding duration or child age, occupation, and medication usage (if any) for each individual.

**Table 2:** Demographic characteristics of mothers

Subject No	Age	Breastfeeding Duration	Medication Usage
1	35	18 months	None
2	30	21 months	None
3	32	20 months	None
4	31	19 months	None
5	28	17 months	None
6	30	23 months	None
7	29	20 months	None
8	32	19.5 months	None
9	30	24 months	None
10	30	15 months	None
11	34	24 months	None
12	34	24 months	None
13	35	10 months	None
14	43	22 months	Eutrox 25 × 1
15	33	24 months	None
16	34	20 months	None

**Fig. 2:**
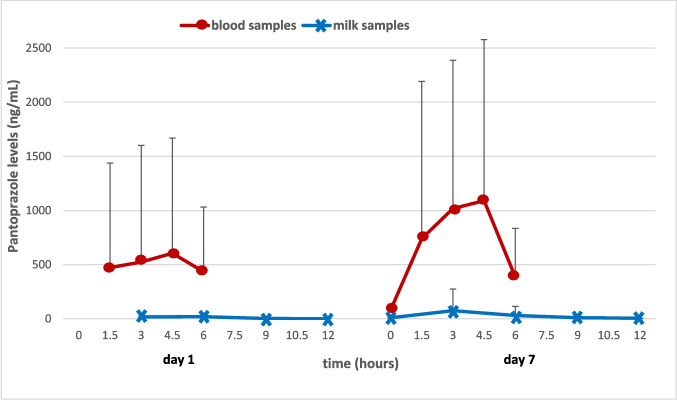
Mean (± standard deviation) Pantoprazole Concentrations in Plasma and Breast Milk. Mean (± standard deviation) pantoprazole concentrations in plasma and breast milk at each sampling time point on day 1 and day 7. Values below LOQ were treated as zero. Pantoprazole concentrations in plasma reached their maximum at approximately 4.5 h on both days, whereas peak levels in breast milk occurred earlier

Analysis of 150 blood samples collected from 16 subjects on days 1 and 7 revealed pantoprazole detection in 46% of samples (69 samples) (Fig. 2, Table 3) (Supplementary Table 1). On day 1, 16 samples were collected, and on day 7, 14 samples were collected. The numbers of detectable samples above the detection limit are indicated in Table 3. Pantoprazole levels in blood peaked at approximately 4.5 h post-administration on both days (Fig. 2), where plasma concentrations were consistently higher than those in breast milk across all time points.

**Table 3:** Pantoprazole Levels in Plasma on Days 1 and 7

		Plasma pantoprazole level (ng/ml)					
	hour	Mean	Std	n	n (> detection limit)	Min	Max
day 1	**0**	0	0	16	0	0	0
	**1.5**	472.3	965.3	16	5	0	3166.2
	**3**	523.9	1076.9	16	6	0	4255.6
	**4.5**	604.7	1064.1	16	8	0	3041.7
	**6**	434.3	596.3	16	11	0	1977.9
day 7	**0**	80.9	0.0	14	1	0	1132.3
	**1.5**	740.4	1451.7	14	5	0	4400.6
	**3**	1017.8	1369.9	14	9	0	5274.9
	**4.5**	1090.3	1487.5	14	12	0	5475.8
	**6**	385.3	448.6	14	12	0	1479.3

(The lower limit of quantification (LOQ) was 0.03 µg/mL. Samples with concentrations below the LOQ were assigned a value of zero for mean calculations)

Analysis of 149 breast milk samples from 16 subjects on days 1 and 7 showed pantoprazole in 17.4% of samples (26 samples) (Fig. 2, Table 4) (Supplementary Table 1). On day 1, 16 samples were collected. On day 7, 14 samples were collected, except for subject 12, for whom insufficient milk volume resulted in a final sample size of 13. Detectable samples above LOQ are indicated in Table 4. Pantoprazole levels peaked at the 3rd–4.5th hours on day 1 and at the 3rd hour on day 7, with detectable concentrations primarily observed at early post-dose time points (Fig. 2). Based on the mean milk concentration observed at day 7 (75.0 ng/mL, corresponding to peak levels at 3 h post-dose), the estimated worst-case infant dose via milk was approximately 0.011 mg/kg/day, assuming a standard milk intake of 150 mL/kg/day. This represents a conservative estimate, as peak milk concentrations are not sustained over the full 24-h dosing interval.

**Table 4:** Pantoprazole Levels in Breast Milk on Days 1 and 7

		Breast milk pantoprazole level (ng/ml)					
	hour	Mean	Std	n	n (> detection limit)	Min	Max
day 1	**0**	0	0	16	0	0	0
	**3**	18.1	36.8	16	5	0	141.0
	**6**	19.6	34.4	16	5	0	104.9
	**9**	2.8	0	16	1	0	45.3
	**12**	0	0	16	1	0	0
day 7	**0**	9.4	0	14	1	0	131.6
	**3**	75.0	200.0	14	6	0	762.5
	**6**	30.2	83.5	14	4	0	315.5
	**9**	10.3	29.5	14	2	0	106.9
	**12**	4.7	0	13	1	0	60.8

(The lower limit of quantification (LOQ) was 0.03 µg/mL. Samples with concentrations below the LOQ were assigned a value of zero for mean calculations)

## Discussion

Little is known about the safety of PPIs during lactation. The current literature on this topic is largely limited to animal studies and review articles, along with a small number of case reports. This study represents the first series involving 16 mothers who discontinued breastfeeding in order to take pantoprazole. The long enrollment period of eight years reflects the difficulty in recruiting such participants. In our study, blood and breast milk samples were collected on days 1 and 7 from mothers who had previously discontinued breastfeeding and were prescribed 40 mg pantoprazole. Blood pantoprazole levels peaked at approximately 4.5 h on both days, whereas breast milk levels peaked at 3–4.5 h on day 1 and at 3 h on day 7.

Although some data exist regarding the use of PPIs during pregnancy [19, 20], the present study focuses specifically on drug transfer into human breast milk during lactation. When considering medications during breastfeeding, it is important to evaluate the potential transfer of the drug into breast milk and its possible impact on the infant. The extent of transfer depends on several factors, including the physicochemical properties of the drug, maternal metabolism, and timing of administration. PPIs are known to pass into breast milk to some extent, but concentrations may vary. Although PPIs are widely used, data regarding their levels in human breast milk remain limited, particularly in lactating women and breastfed infants. For example, a case report demonstrated pantoprazole excretion into breast milk with a milk/plasma ratio of 0.022, measured 2 h after administration of a 40 mg dose in one woman monitored for 24 h [12]; however, this finding is limited by the fact that the ratio was assessed at only a single time point. Another report on esomeprazole showed that it was undetectable in breast milk at 10 h post-dose and absent in the infant’s serum at 12 h [21]. Marshall et al. reported peak omeprazole concentrations in breast milk of 58 nM (approximately 22 ng/mL) (3 h post-dose), which represented less than 7% of the peak serum concentration (950 nM at 4 h) in a breastfeeding mother receiving 20 mg/day [22]. In our study, pantoprazole was infrequently detectable in breast milk, and milk concentrations were consistently lower than plasma concentrations, suggesting limited transfer into breast milk. This relatively low ratio indicates limited transfer of pantoprazole into breast milk. In addition to the milk-to-plasma ratio, we estimated infant exposure based on observed milk concentrations. It should be noted that this estimate represents a worst-case scenario, as it assumes that peak milk concentrations are maintained throughout the entire 24-h dosing interval, which is not physiologically realistic. In practice, milk concentrations decline after peak levels, and overall infant exposure is expected to be lower. To further contextualize exposure, a theoretical maternal weight-normalized dose was estimated assuming a standard body weight of 70 kg (equivalent to 0.57 mg/kg/day for a 40 mg dose). Based on this, the estimated worst-case RID was approximately 1.9%, which is well below the commonly accepted safety threshold of 10% [23]. Using the mean peak milk concentration at day 7 (75.0 ng/mL at 3 h post-dose) and a standard milk intake assumption of 150 mL/kg/day, the estimated infant dose via milk was approximately 0.011 mg/kg/day. This low estimated exposure further supports the limited transfer of pantoprazole into breast milk.

This study has several limitations that should be considered. Recruitment of participants was challenging and spanned eight years, reflecting the difficulty in identifying mothers who had decided to discontinue breastfeeding while maintaining active lactation. Measurements were conducted across three time periods, which may introduce variability despite the use of consistent analytical methods; this extended timeline was partly influenced by interruptions to clinical research during the COVID-19 pandemic.

Second, the sample size was limited (n = 16), and the relatively low detectability of pantoprazole in plasma and especially breast milk samples may reflect both pharmacokinetic properties of the drug and analytical sensitivity, which should be considered when interpreting the findings. Additionally, maternal body weight was not prospectively recorded; therefore, maternal dose could not be normalized to mg/kg/day and formal RID calculation was not feasible, limiting comparison with established safety thresholds.

Furthermore, the absence of infant serum measurements or clinical outcome data, together with the inclusion of mothers in the late lactation period (14–28 months postpartum), during which milk production may vary and be lower than in early lactation, may limit the generalizability of the findings.

In summary, pantoprazole levels in plasma peaked at approximately 4.5 h and declined significantly thereafter, whereas concentrations in breast milk appeared to decrease after the 3rd hour. Pantoprazole levels were consistently lower in milk than in plasma and were infrequently detectable. The estimated infant dose via milk was approximately 0.011 mg/kg/day, indicating low infant exposure. This series represents the largest dataset to date evaluating pantoprazole excretion during lactation.

## Conclusion

Pantoprazole appears to be minimally excreted into human breast milk, with infrequent detectability and consistently lower concentrations in milk than in plasma. The estimated infant dose via milk was approximately 0.011 mg/kg/day, suggesting low infant exposure. The estimated worst-case infant dose via milk was approximately 0.011 mg/kg/day, corresponding to a worst-case RID of approximately 1.9%, which is well below the commonly accepted 10% safety threshold. However, because formal RID calculation was not possible and no infant clinical outcome data were available, these findings should be interpreted with caution. Further studies including maternal body weight data and comprehensive pharmacokinetic evaluation are warranted. This study provides pharmacokinetic data that may help inform clinical decision-making for lactating mothers requiring pantoprazole therapy.

## Supplementary Information

Below is the link to the electronic supplementary material.

Supplementary file1 (XLSX 12 KB)

## Data Availability

The datasets generated and analysed during the current study, including pantoprazole plasma and breast milk concentration values and analytical method validation data, are included in this published article and its supplementary information files.
